# Progress of Surgery

**Published:** 1903-07-25

**Authors:** 


*(To be continued.)*

### PROGRESS OF SURGERY.

### GENITO-URINARY.

**Cystoscopy.**—John G. Pardoe[1] has contributed a paper pointing out the value of the cystoscope in the diagnosis of diseases of the urinary system and recording some half-dozen cases which illustrate the value of the instrument in a striking way. The writer says that the interest taken in cystoscopy by the majority of surgeons is very faint, and that the text-books on surgery in the English language do not strongly urge the importance of the procedure and the valuable results to be obtained with it. Case 1 was that of a man who had had for some months intermittent hæmaturia, abnormal frequency of micturition, and pain in the supra-pubic and other regions. The bladder had been sounded many times with negative results. Cystoscopy showed a normal bladder with the exception that the right ureteral orifice was swollen and the gleam of a calculus lodged in it was seen. The patient was directed to drink large quantities of bland fluid and to take violent exercise. Three days later he passed a small calculus. Thus the provisional diagnosis of a growth in the bladder was given up and suprapubic cystotomy avoided. Case 2 was that of a man who for six months had had severe pain in the left lumbar region, intermitting in character and accompanied by hæmaturia. Between the attacks the urine was normal. The cystoscope showed a villous growth attached by a pedicle to the bladder-wall near the left ureter. This was removed supra-pubically, and a useless nephrotomy was avoided. Case 3 was that of a man with enlarged prostate who had been treated by litholapaxy three times, had been entirely dependent on his catheter for nine months, and had bad cystitis. The cystoscope showed a stone, 1 inch in length, lying under a pendulous median enlargement of the prostate. The latter was attached by a pedicle to the main part of the prostate. Both the pendulous growth and the stone were removed through a small perineal incision and the cystitis cured by drainage. The wound healed in a fortnight. Thus, by the aid of cystoscopy, a simple operation was substituted for a much more dangerous one and convalescence shortened. In Case 4 the cause of four or five attacks of profuse hæmaturia during a period of nine months was shown by cystoscopy to be a large single ulcer near the left ureteral orifice. Tubercle bacilli were afterwards found in the urine. Under treatment the ulcer healed. The diagnosis could hardly have been established without cystoscopy except by operation, which latter was thus avoided. Any objections which can be urged against cystoscopy apply equally to other metal instruments of a like calibre.

**The Urine from each Kidney.**—F. C. Valentine, New York,[2] has recently described Cathelin's instrument for separately collecting the urine from each kidney and the method of using it. He regards the methods of Harris and Downes as applicable essentially in the female, and he is disposed to think that

Kelly's and Paulik's methods are better. Ureteral catheterism, with the cystoscopes of Nitze, Casper, or Albarran, is the best method of obtaining the urines separately, but it has its limitations. For catheterization the bladder must have a capacity of at least 50 c.c., there must be no bleeding, and the ureters must be large enough to admit the smallest catheters. Cathelin separates the urines in the bladder with a divisor. Lambotte, of Brussels, in 1890, first made use of a similar method. Cathelin's graduated vesical divisor is a tube shaped like a short-beaked catheter, which is traversed by a central and two lateral channels. Its calibre is 25 in the French catheter scale. The central channel is for the conduction of a shaft with a graduated handle. This shaft is pushed into its channel until it projects at the distal central orifice at the base of the beak of the instrument on its convex under surface. The projecting part is then fixed by means of a small spring to a soft rubber membrane set within a delicate steel spring. The rubber membrane can be then withdrawn entirely into the tube by means of the shaft, or protruded again at will. The scale on the shaft indicates how far the membrane has to be protruded into the bladder according to the previously estimated minimal capacity of the bladder. This vesical capacity is measured by noting how much fluid has to be injected slowly into the empty bladder to cause a desire to urinate. The two lateral channels in the instrument transmit catheters which can be projected into the bladder on each side of the rubber membrane. The projected membrane lies vertically in the bladder, and adapts itself to all the irregularities of the floor of the bladder. The catheters then syphon off the urine which collects on each side of the rubber partition. Valentine describes exactly how the instrument is used and sterilised.

**The Surgical Treatment of Anuria.**—A. Dean Bevan, of Chicago,[3] has contributed a paper with this title. He points out that the subject has not received the attention it deserves, that most modern text-books on surgery dismiss it with brief consideration, and few practitioners are familiar with it. The recognition of the condition, however, is very important because there is a wide difference between the results when it is recognised and adequately treated and when it is ignorantly treated. Seventy-five per cent. of cases recover if operated upon early, whereas but 25 per cent. survive if not operated upon. The author suggests the clinical grouping of cases of suppression of urine into three classes, viz., the obstructive, the reflex or paralytic and the non-obstructive. The *obstructive class* includes those in which the obstruction is of the ureter of a single functioning kidney, the other kidney being either absent congenitally or destroyed by disease, and also cases in which both ureters become obstructed simultaneously. The *reflex or paralytic class* includes, (*a*) obstruction of one ureter causing suppression of function in the opposite kidney; (*b*) removal of one kidney acting similarly; (*c*) hysterical anuria, and (*d*) trauma of one or both kidneys. *Non-obstructive Anuria* arises from nephritis due to fevers, anæsthetics, poisons, such as phosphorus and turpentine, toxins in general diseases, *e.g.*, cholera, urethral fever, and lesions which gradually destroy first one and then the other kidney—such as tuberculosis or cystic degeneration.

Anuria is merely a condition, not a disease. Uræmia is a disease, a toxæmia developing from nephritis or in the course of anuria. Patients with anuria may continue in fair condition for a week or even two weeks without uræmic symptoms developing. Uræmic patients seldom live more than three days after the symptoms begin, with total suppression co-existing. Uræmia is the usual cause of death in anuria. In obstructive anuria there is usually pain on the side last obstructed, and tenderness and muscular rigidity are usually found also. The imperative treatment is nephrotomy on the kidney last attacked not later than 48 hours after the onset of the symptoms, and the symptoms mentioned above usually show clearly which side has to be operated upon. Nitrous-oxide anæsthesia is recommended as the safest for the procedure, and no prolonged operation should be performed. The existence of reflex anuria has been denied by many, but Bevan believes it may occur but that it is rare. In operating for the relief of reflex anuria the sound kidney should not be touched. For example, if a hydro-nephrotic kidney is causing reflex anuria the tension in the diseased kidney should be relieved. But practically there will usually be great difficulty in deciding which kidney is to be operated upon, because the diagnosis as to whether the anuria is reflex or not cannot be made with certainty. The author therefore recommends that if an operation on the side yielding the symptoms of pain and tenderness fails to relieve the anuria in 24 hours, then nephrotomy should be performed on the other side. Anuria from traumatism of one or both kidneys should be treated by draining the kidney. With regard to nephritic anuria a number of clinical results seem to justify operation on the kidneys for the relief of congestion. Thus Israel advocates nephrotomy on one kidney in anuria from scarlet fever nephritis.

[1] Lancet, March 28, 1903, p. 870. [2] Zentralb. f. Chir., Jan. 17, 1903, p. 92. [3] Ann. of Surg., April, 1903, p. 575.

